# Perceived role of digital health technologies for facilitating diabetes self-management and education among low-income adults with type 2 diabetes: a qualitative needs assessment to inform support for newly diagnosed adults

**DOI:** 10.1186/s12889-026-28880-2

**Published:** 2026-08-11

**Authors:** Advait Rajan, Michelle D. S. Boakye, Anthony I. Ajayi, Gabriel Q. Shaibi, Eyitayo O. Owolabi

**Affiliations:** 1 Center for Health Promotion and Disease Prevention, Edson College of Nursing and Health Innovation, Arizona State University, 500 N 3rd Street, Phoenix, Arizona 85004 USA; 2 Manning College of Nursing and Health Sciences, University of Massachusetts Boston, Boston, USA; 3 Sexual Reproductive Maternal Newborn Child and Adolescent Health Unit, African Population and Health Research Centre, Manga Close, Nairobi, Kenya

**Keywords:** Digital health, Diabetes self-management behaviors, DSMES, Barriers and facilitators, Low-income, Newly diagnosed

## Abstract

**Background:**

Low-income adults with type 2 diabetes (T2D) face significant challenges engaging in self-management behaviors (SMBs), including physical activity, healthy eating, and medication adherence. Diabetes self-management education and support (DSMES) programs can promote SMBs, but participating in DSMES is often challenging. Digital health technologies may help address barriers to SMB and DSMES engagement. However, evidence remains limited among adults newly diagnosed with T2D, who may experience unique psychosocial challenges, and varying readiness for behavioral change. This study explored digital technologies perceived as desirable and relevant for promoting SMB and DSMES engagement among low-income adults newly diagnosed with T2D.

**Methods:**

We employed an exploratory qualitative design and conducted 27 semi-structured interviews with 23 low-income adults with T2D (15 females, 8 males) and 4 healthcare stakeholders involved in T2D care (3 registered dietitians and 1 registered nurse; all female). Data were analyzed using deductive and inductive thematic approaches.

**Results:**

Participants with T2D ranged in age from 22 to 76 years (mean: 44±18), and all earned <$50,000/year. Only 7 of 23 adults had attended any form of diabetes education. Three major themes emerged regarding the role of digital health: 1) digital health enables flexible access to DSMES, 2) barriers to adoption exist at individual, technological, and system levels, and 3) there is a need for user-centered digital health solutions. Both individuals with T2D and healthcare stakeholders highlighted the importance of hybrid DSMES models that combine in-person education with synchronous and asynchronous digital formats to improve access, flexibility, and ongoing support during the early post-diagnosis period. Health monitoring technologies, including continuous glucose monitors and diet-and activity-tracking apps, were identified as important facilitators of behavioral change. Participants and stakeholders also emphasized the value of integrating digital tools and data visualization into in-person DSMES to strengthen clinician interaction and ongoing engagement. Key barriers highlighted include socioeconomic constraints, limited digital literacy, complex interfaces, and poor integration into clinical workflow.

**Conclusion:**

Digital health technologies that provide synchronous and asynchronous education, remote monitoring, enhanced communication, data visualization, and ongoing support may improve access to DSMES and strengthen engagement in SMBs among low-income adults newly diagnosed with T2D. Hybrid, user-centered approaches that integrate digital tools with in-person DSMES may help address the psychosocial, structural, and technological barriers to sustained diabetes self-management.

**Supplementary Information:**

The online version contains supplementary material available at https://doi.org/10.1186/s12889-026-28880-2.

## Introduction

Despite advancements in medical therapeutics, type 2 Diabetes (T2D) remains a major global health challenge associated with significant morbidity, premature mortality, and increased healthcare costs [1]. According to the International Diabetes Federation, approximately 589 million adults were living with diabetes globally in 2024, with a projected increase to 853 million by 2050 [2]. In the United States (US), an estimated 38.4 million adults currently live with diabetes, more than 90% of whom have type 2 diabetes T2D, and approximately 1.4 million new cases are diagnosed annually [3]. The burden of T2D disproportionately affects low-income populations, who often experience poorer health outcomes due to reduced healthcare access, financial constraints, and broader social inequities [4, 5].

Management of T2D requires engagement in multiple self-management behaviors (SMBs), including medication adherence, dietary modification, regular physical activity, and glucose monitoring. Low-income adults with T2D face substantial barriers to implementing SMBs, including competing demands for time and attention, limited knowledge and self-management skills, and social determinants of health that constrain access to necessary resources and healthcare services [6]. Diabetes Self-Management Education and Support (DSMES) programs are evidence-based interventions designed to address these challenges by providing the education, behavioral skills training, and ongoing support needed to effectively manage T2D from diagnosis onward [7]. However, participation in DSMES remains consistently low. Within the first year of diagnosis, only 6.8% of privately insured individuals with T2D and 5% of Medicare/Medicaid beneficiaries participate in DSMES programs [7, 8]. Underutilization of DSMES has been attributed to logistical and psychosocial barriers, including cost, scheduling conflicts, stigma, and negative perceptions about DSMES [7]. Health system and programmatic barriers, such as providers’ lack of awareness and referral, also exist [7]. These persistent barriers underscore the need for strategies that improve timely engagement in DSMES and support sustained participation in SMBs.

Digital health technologies offer promising opportunities to address barriers to engagement among individuals newly diagnosed with T2D in both SMBs and DSMES. Smartphone ownership has increased substantially across the United States of America (USA), with approximately 61–95% of adults, including many individuals from lower socioeconomic groups and older adults, now owning a smartphone [9]. Digital tools such as mobile applications, telehealth platforms, wearable devices, and text messaging may support self-management outside traditional clinical settings [10, 11]. For example, continuous glucose monitors (CGMs) can help individuals interpret glucose patterns throughout the day [10, 12], while mobile applications can support tracking of physical activity, dietary intake, and other self-management behaviors [13]. Telehealth platforms may also improve access to DSMES by reducing transportation, and scheduling barriers [10]. However, effective implementation of digital health interventions requires approaches that are responsive to the preferences, lived experiences, and contextual needs of adults newly diagnosed with T2D.

While there is extensive literature on the potential of digital health technologies to support both SMBs and DSMES, evidence on their effectiveness remains mixed and is limited among adults newly diagnosed with T2D. For example, a systematic review and meta-analysis reported that smartphone applications and short message service (SMS) interventions, but not website-based interventions, were associated with improved glycemic control in individuals with T2D [14]. However, the review did not specifically examine outcomes among individuals newly diagnosed with T2D. Adults newly diagnosed with T2D may experience psychosocial challenges and varying levels of readiness for behavioral change that influence engagement in DSMES, self-management, and digital health support [15–17]. A recent scoping review identified emerging evidence supporting the use of digital health technologies for self-management among those newly diagnosed with T2D, although most studies were conducted outside the USA [15]. The review additionally emphasized the importance of aligning digital health approaches with patients’ preferences, contexts, and lived experiences to maximize engagement and impact.

Given the scarcity of evidence for low-income adults newly diagnosed with T2D, this qualitative needs assessment sought to identify digital technologies perceived as desirable and relevant for promoting engagement in SMBs and DSMES for this population. To inform intervention development and in response to recommendations from community stakeholders, we included perspectives from adults living with T2D across the disease trajectory, asking participants to reflect on their experiences, challenges, and support needs at the time of diagnosis. This approach enabled us to draw on the retrospective insights that come with living with T2D over time while maintaining a focus on the newly diagnosed experience. To better contextualize the findings, we also explored barriers and facilitators to DSMES engagement in our study context from the perspectives of both individuals with T2D and healthcare professionals.

## Methods

### Study design

This study employed an exploratory qualitative design, utilizing semi-structured individual interviews to understand the role of digital health technologies in facilitating engagement in SMBs and DSMES with for low-income adults newly diagnosed with T2D. The study addressed the following research questions: (1) What barriers and facilitators influence engagement in DSMES within our context? (2) What roles might digital health technologies play in facilitating engagement in DSMES and SMB? and (3) Which digital health technologies do low-income adults with T2D perceive as desirable and relevant for supporting DSMES and SMB when newly diagnosed with T2D? The study reporting followed the Consolidated Criteria for Reporting Qualitative Research (COREQ) guideline (Supplementary Material 3) [18].

### Research team and reflexivity

The research team consisted of male and female researchers who shared an interest in addressing T2D disparities through community engagement, intervention development, and evaluation. The principal investigator (PI; last author) and the second author have nursing backgrounds and extensive experience conducting research with individuals living with T2D. Their approach to data collection, analysis, and interpretation was informed by an interest in identifying opportunities for intervention targeting adults newly diagnosed with T2D. The PI (EOO) conducted all interviews. The first author (AR) and the PI (EOO) led data transcription, coding, analysis, and interpretation, with input from authors MDSB and AIA. AR is an undergraduate minority student with personal experience with family members living with T2D and a strong interest in diabetes disparities and digital health technologies, which informed his interpretation and presentation of the findings. He brought a unique perspective to the data and its interpretation. AIA is a social scientist with extensive experience designing, analyzing, and reporting qualitative research. GQS is a senior researcher with expertise in T2D disparities and evidence-based intervention development. Collectively, the research team brought diverse disciplinary perspectives and lived experiences to the interpretation and presentation of the findings.

The research team acknowledges that the broader goal of using the study findings to inform future intervention development may have influenced the interpretation of the data. To enhance rigor and minimize potential bias, multiple researchers participated in data analysis and interpretation, and findings were reviewed through iterative discussions and interpretation checks with participants. Additionally, the perceptions of healthcare stakeholders regarding the required technologies may not accurately reflect what individuals with T2D truly desire.

### Participants and sampling

Study participants included adults (≥ 18 years) with T2D who were in the low-income category, defined as eligible for/receiving the Supplemental Nutrition Assistance Program, uninsured, Medicaid beneficiaries, or with an annual income of less than $50,000. In addition, we included healthcare providers involved in diabetes care (thereafter referred to as “stakeholders”). Participants were purposively recruited across Phoenix, Arizona, through three avenues: Arizona State University’s online research participant registry (Research PlusMe), a community clinic, and a food pantry. We placed study flyers at the different locations, including the online space. Sample size was guided by the principle of data saturation, defined as the point at which additional interviews yielded no substantially new insights relevant to the study questions [19]. Twenty-nine individuals expressed interest in participating; of these, 27 individuals (23 adults with T2D and 4 healthcare stakeholders) met the eligibility criteria and completed interviews. The stakeholder group included three registered dietitians/diabetes care and education specialists and one registered nurse involved in DSMES programs serving low-income individuals across different healthcare settings.

Although the study focused on adults newly diagnosed with T2D, individuals with longer diabetes duration were included to capture broader perspectives on DSMES engagement and digital health use across the disease trajectory. Interview questions specifically prompted participants to reflect on their experiences and needs at the time of diagnosis. Ten of the 23 adults with T2D had been diagnosed within the previous 24 months. The inclusion of individuals with longer diabetes duration was also informed by recommendations from community stakeholders, who emphasized the importance of incorporating diverse lived experiences and perspectives.

### Data collection

Semi-structured individual interviews were conducted to explore the study’s research questions. Interviews were conducted either in person or virtually via Zoom, based on participants’ preferences. In-person interviews took place in a private space within a community-based food pantry. Only the participant and members of the study team were present during interviews. No participants withdrew from the study after enrollment, and repeat interviews were not conducted.

The study team developed separate interview guides for adults with T2D (Supplementary File 1) and healthcare stakeholders (Supplementary File 2) based on the study objectives. Patient interviews focused on their experiences managing T2D, engagement in SMB and DSMES, perceived barriers and facilitators to engagement, the potential role of digital health technologies, and lastly, the type(s) of digital health interventions they would need to better manage their T2D as a low-income person newly diagnosed with T2D. The stakeholder interview guide addressed similar topics but was adapted to reflect their professional roles and experiences in diabetes care delivery. The interview guides were pilot tested with individuals not included in the study and subsequently refined by the research team. Interviews lasted approximately 45–60 min and were conducted in English by the last author. All interviews were audio-recorded, and field notes were documented during data collection. Data collection took place between February and August 2024.

### Ethical considerations

Ethical approval was obtained from the Arizona State University Institutional Review Board in accordance with the Declaration of Helsinki II. All participants provided informed consent prior to participation. All data were kept confidential and anonymous. Verbatim quotations were deidentified and assigned identification numbers along with limited relevant socio-demographic characteristics where appropriate.

### Data analysis

The interviews were transcribed verbatim by a professional transcriber and a trained undergraduate research assistant (AR-male). Transcripts were reviewed for accuracy and imported into NVivo 15 qualitative analysis software (Lumivero, Colorado, USA). Data analysis followed the six steps of thematic analysis highlighted by Braun and Clarke [20]. First, we read the transcripts a few times for familiarization. This was followed by initial code generation, theme identification, theme review, theme refinement and naming, and finally, writing the report. The PI (EOO-female) and the first author (AR) conducted the initial analysis of all transcripts and coded the data line by line. Initial codes were then shared with MDSB (female), and the authors met to discuss, develop, and refine the relevant themes and subthemes. AIA (male) contributed to the refinement of the final codes, themes, and subthemes. GQS (male) provided expert input on the interpretation and presentation of the findings. Data analysis used both inductive and deductive approaches, guided by the study objectives, while allowing new concepts to emerge from the data.

### Trustworthiness and rigor

Several strategies were employed to enhance the trustworthiness and rigor of the study. Credibility was strengthened through investigator triangulation, with three members of the research team involved in data analysis and interpretation. The investigators met regularly to discuss codes, share perspectives, compare interpretations, and refine themes to support accurate and comprehensive data interpretation. Credibility was further enhanced through triangulation of perspectives across diverse participant groups, including adults with T2D and healthcare stakeholders. We conducted member checking by sharing the results and discussion draft with some participants to ensure that interpretations accurately reflected their experiences and perspectives. Transferability was supported through a detailed description of the study context, participant characteristics, data collection procedures, and analytic processes [21].

### Findings

Table 1 summarizes participants’ demographic characteristics. A total of 27 individual interviews were conducted, including 23 low-income adults with T2D and 4 healthcare stakeholders. Of the 23 individuals with T2D, 15 were female, 9 were Medicaid or Medicare beneficiaries, and 8 were uninsured. Participants ranged in age from 22 to 76 years, and the duration since diagnosis ranged from 1 to 20 years. Only 7 of 23 adults with T2D had attended either a formal or informal diabetes education program. The stakeholder group consisted entirely of female participants, including three registered dietitians/diabetes care and education specialists and one registered nurse. Their professional practice duration ranged from 10 to 72 months.

**Table 1:** Sociodemographic characteristics of individuals with type 2 diabetes and stakeholders

Socio-demographic characteristics	Low-income individuals with T2D *N* = 23	Health stakeholders *N* = 4
Age in years Mean (Range)	44 (22–76)	41 (26–69)
Duration of diabetes in months, mean (range)	5 (1–20)*	-
Duration of practice in months, mean (range)	-	43 (10–72)
Sex	Female (*n* = 15)	4
	Male (*n* = 8)	0
Education level	Graduate degrees (*n* = 2)	1
	College (*n* = 12)	3
	High school-some college (*n* = 9)	-
Insurance status	Medicaid/Medicare beneficiaries (*n* = 9)	-
	Uninsured/self-pay (*n* = 8)	-
	Private (*n* = 6)	-
Average annual income	$0–19,999 (*n* = 7)	-
	$20,000–49,999 (*n* = 16)	-
Race/ethnicity	Hispanic/Latino (*n* = 8)	3
	African American/Blacks (*n* = 11)	-
	American Indian (*n* = 1)	-
	Unreported (*n* = 2)	-
	Non-Hispanic white (*n* = 1)	1
Comorbidity/comorbidities?^#^	Yes (*n* = 9)	-
	No (*n* = 14)	-
Attended a diabetes education class?	Yes (*n* = 7)	-
	No (*n* = 16)	-
Designation/job title	-	Registered Dietitian (*n* = 3)
	-	Registered Nurse (*n* = 1)

*10 of 23 participants were diagnosed in the past 1–2 years

^#^Comorbidities included fatty liver disease (*n* = 1), metabolic syndrome (*n* = 1), lupus (*n* = 2), rheumatoid arthritis (*n* = 3), hypertension (*n* = 1), neuropathy (*n* = 1), osteopenia (*n* = 1), binge eating disorder (*n* = 1), and polycystic ovarian syndrome (*n* = 1). Some participants reported multiple comorbidities

### Barriers and facilitators to DSMES engagement

Barriers and facilitators to DSMES engagement are presented under two major themes: 1) individual/patient-level factors and 2) organizational/programmatic factors.

#### Theme 1: individual/patient-level factors

##### Subtheme 1a: competing life demands and immigration-related challenges

Participants noted that their personal circumstances and daily life demands constrained their ability to engage in DSMES. Engaging in DSMES was often described as a lower priority amid several other responsibilities. Greater priority is given to employment, spending time with family, and other household responsibilities, such as cooking and childcare. These activities not only limited participants’ time availability but could also be exhausting, leaving no room for DSMES attendance.

*“…I think I’ve kind of heard about those classes [DSMES]*,* but yeah*,* it’s one of those things you just brush off because you’re so busy with everything else. You have work*,* you have life*,* you have everything else you need to deal with. So*,* like*,* you don’t think about the importance of what that can do for you.”* (PWD1, never attended DSMES)

Immigration status was another notable obstacle to DSMES participation cited by both patients and stakeholders. Participants reported that fear of discrimination and deportation could discourage engagement in in-person DSMES classes even when the need for support is apparent. In addition, being undocumented resulted in a lack of insurance coverage, requiring out-of-pocket fees for DSMES classes. These factors discourage DSMES attendance:

*“The status*,* their documentation status*,* can also be a barrier. I think there’s fear of potential to get*,* you know*,* deported*,* things like that*,* and so they choose not to get that care [DSMES].”* (Stakeholder 3)

Some facilitating factors that could address these barriers were mentioned. These included flexible class delivery formats that accommodate life commitments, minimizing out-of-pocket costs, and screening for and addressing social needs such as transportation and childcare support. Some also suggested including family members in the DSMES classes, noting that this could make the classes more engaging and impactful while creating a more welcoming environment for individuals with children or caregiving responsibilities.

##### Subtheme 1b: psychological and cognitive factors

Participants alluded to some psychological barriers impacting DSMES engagement. They noted that individuals newly diagnosed with T2D may experience different psychological issues at the time of diagnosis, including being in denial or having apathy or anxiety, which can impact their willingness and readiness to take charge of their T2D management, including engagement in SMBs and DSMES.

“*I would say immediately after I was diagnosed. I was a little anxious about it*,* so it took me some time to put my mind to it and come to terms with the whole concept of*,* “Oh*,* you’re diabetic.” It took me some time. Like I said*,* mental health is very important*,* and I needed to put myself together before venturing into any of these classes*.” (PWD2, never attended DSMES)

Others pointed to the impact of the negative perceptions of DSMES on their willingness to participate in the class. Some participants believed that DSMEs would offer little beyond generic dietary education, would be overly didactic and “boring”, and would not be worth their time. These findings aligned with reports of limited understanding of the benefits of DSMES for T2D self-management.

*“I’m not a very busy person*,* but that just seems like a lot of time I’m going to waste*,* like I told the other dietitian doctor*,* I do what I do… You are not going to get more exercise out of me than what I’m doing right now. Nothing you are going to tell me is going to make me do anything more or less than what I’m doing right now. It seems I would just be sitting there bored. I think it’ll be a waste of time.”* (PWD3, never attended DSMES)

Participants also highlighted ways to address the negative perceptions of DSMES. They emphasized the need for DSMES educators to develop creative educational materials and outreach strategies that clearly communicate the purpose, benefits, and content of DSMES through advertisements and testimonials. In addition, they stressed the importance of recommendations from their primary care providers, who are often trusted sources of information and can help validate the value of participating in DSMES.

*“If the program is well-advertised and the purpose is clear*,* it would be appealing. It should be for everyone*,* not just those diagnosed*,* so they can extend the knowledge to others.”* (PWD4, never attended DSMES)

#### Theme 2: organizational/programmatic barriers

##### Subtheme 2a: lack of referral

Despite the well-documented health and psychosocial benefits of DSMES, particularly for individuals newly diagnosed with T2D, both patients and the stakeholders identified lack of referral as a prominent programmatic barrier. Due to a lack of referrals, most study participants were unaware of DSMES programs and often relied on less formal or unvalidated sources of information, such as YouTube, TikTok, and other social media platforms, following diagnosis. Many participants noted that they would have attended DSMES classes had they been informed about and referred to them earlier in their diagnosis journey.

*“Yes*,* it would have been nice to have that offered at the beginning [when first diagnosed with T2D]. I feel like I should ask for it*,* but if it had been offered*,* I would have attended.”* (PWD5, never attended DSMES)

Stakeholders also described challenges related to providers’ lack of clarity and misconceptions regarding DSMES eligibility and referral processes. Some providers were reportedly uncertain about DSMES eligibility criteria, how to identify individuals who qualify for referral, or how to initiate referrals within their electronic health record system.

*“So I think one of the challenges is the initial referral to the program. One is getting providers to actually identify and send the appropriate referral.”* [Stakeholder 3]

##### Subtheme 2b: program delivery, content, and format

Additional program-related barriers were associated with program design, delivery, and content. Participants identified the timing of DSMES classes as a barrier to engagement, noting that most classes are scheduled during working hours and that evening options are limited. That, coupled with family responsibilities as highlighted under individual-level challenges, constitutes a barrier to engagement in DSMES.

“Classes… are usually in the morning when I’m working. That makes it hard to enroll.” (PWD5, never attended DSMES)

Participants also reported that the predominantly didactic nature of most DSMES classes, limited interactive sessions, and excessive use of technical medical jargon made the content difficult to comprehend and less engaging. They suggested that DSMES should extend beyond generic diabetes education to incorporate more hands-on and practical learning. Participants further emphasized that educators should use the first class to create a positive and motivating experience that encourages continued participation. A positive and welcoming attitude from educators was equally viewed as important for engagement.

“So I think that the person providing the DSMES can really just, through their magnetic personality, gather some compliance. I think that once people start receiving information they can work with, they will understand. It might feed a desire to have more because they become more familiar with the content and how it’s being presented, and it seems less mysterious and frightening.” (Stakeholder 2).

A lack of ongoing support, engagement, and communication from educators and primary care providers between DSMES sessions was also noted as a program-related barrier, particularly regarding retention. Stakeholders noted considerable gaps between DSMES sessions for low-income individuals, with few opportunities for follow-up, reinforcement, or communication between classes. As a result, some patients lacked support in applying newly acquired knowledge, receiving feedback, or addressing emerging questions, thereby reducing the perceived value and impact of DSMES participation. Stakeholders further noted that limited infrastructure for ongoing support, coupled with already overburdened healthcare systems and providers, contributed to these challenges.

“Oftentimes, these patients are only seen every three months until their primary care provider visit. Within these three months, there are just so many good opportunities to provide diabetes education, but many of these patients are not getting that consistent follow-up. So, they come back and, you know, their A1C is the same.” (Stakeholder 4)

### The role of digital health technologies for facilitating engagement in DSMES and SMBs

Participants identified a spectrum of digital health technologies they considered relevant for T2D self-management and DSMES engagement. These findings are presented under three themes: (1) digital health enables flexible access to DSMES, (2) barriers to adoption exist at individual, technological, and system levels, and (3) the need for user-centered digital health solutions.

#### Theme 1: digital health enables flexible access to DSMES

Participants widely acknowledged the limitations of traditional in-person DSMES, especially the logistical hurdles and time constraints, and described the potential of digital health technologies to provide greater flexibility and convenience. Specifically, challenges related to class scheduling and competing demands made the flexibility and convenience offered by digital platforms highly appealing. Participants expressed preferences for both synchronous online and asynchronous on-demand educational platforms. As highlighted in Table 2, participants noted that platforms offering synchronous and asynchronous learning could incorporate multimodal features such as video, audio, testimonials, and structured text to enhance learning.

“.if it was something I could do online… I would take the time to do it, but as far as having to go somewhere else… I don’t think I’ll do it” (PWD6, never attended DSMES, owns a smartphone, attended the interview via Zoom)

“if it’s something I can do myself when I have time… not being rushed… I kind of like stuff like that” (PWD3, never attended DSMES, owns a smartphone, attended the interview via Zoom)

Participants also identified the need for improved patient-provider communication, ongoing support, and follow-up between DSMES appointments as important facilitators of SMB and DSMES engagement. They described these support mechanisms as often nonexistent or suboptimal and expressed interest in digital communication platforms that would allow ongoing engagement with healthcare providers and educators, as well as the opportunities to receive feedback on health data. They viewed these communication features as addressing the previously identified barrier of limited continued support.

“I need to have a certain way to contact my doctor and let her know this is going on, not wait until three months later…” (PWD7, never attended DSMES, owns a smartphone, attended the interview via Zoom)

Regarding enhanced engagement in SMB, participants expressed considerable interest in health-monitoring tools, especially continuous glucose monitors (CGMs). Self-monitoring is a key SMB and component of the DSMES classes. Participants described CGMs as convenient tools for blood glucose monitoring that enhanced understanding, motivation, and problem-solving related to lifestyle behaviors and glucose patterns. Some participants also valued the ability to monitor additional health data, including diet, physical activity, and sleep. These monitoring preferences closely aligned with participants’ desire for ongoing communication and feedback from healthcare providers. One participant described intermittent CGM use due to financial constraints as a “self-invented intervention,” explaining how periodic monitoring helped him better understand the relationship between lifestyle habits and blood glucose control.

“Like this doctor at the clinic, I told him that it’s [CGM] really the best thing for me. It works better than anything else. And he said, Insurance doesn’t pay for it. And I said, I know, I pay for it in cash. I use it for just two weeks, and then I wait for four weeks before using another one, as it helps me monitor my habits. It might even be my self-invented intervention to do it like that, but I think it’s a really, really good intervention because it helps me play a game while I have it on. And when I have it off.” (PWD8, never attended DSMES, owns a smartphone, attended the interview via Zoom, a prior CGM user)

Although participants largely described CGMs as motivating and empowering tools for self-management, one stakeholder expressed concern that continuous exposure to glucose data could become overwhelming or anxiety-provoking for some individuals. This difference in perspectives may reflect differing assumptions among healthcare providers and patients about the emotional impact and usability of continuous self-monitoring technologies.

“I feel like a continuous glucometer could be a little bit more overwhelming for a lot of people, just because they’re seeing the sugar levels constantly, and I feel like that can stress people out. As it is, I do have a few patients who are like, I don’t wanna check my sugar levels every day because it stresses me out.” (Stakeholder 1)

Another stakeholder, however, noted that features generated through health monitoring technologies, such as graphical data visualizations, could enhance DSMES delivery by improving patient understanding and making classes more interactive and engaging, thereby addressing the negative perceptions that DSMES classes can be “boring”.

Some participants additionally expressed interest in multifaceted digital platforms that combine data tracking and monitoring, communication, reminders, social support, and on-demand education. When asked what features they would like to see in a program designed to enhance self-management for newly diagnosed, a participant described:

“I think maybe an app that helps me– that can remind me when to take my medications. An app that can help me in the aspect of exercise, an app that can remind me when to exercise and can remind me how to exercise, and an app that can help me actually monitor my sugar level and everything … and my food.” (PWD9, never attended DSMES, owns a smartphone and attended the interview via Zoom)

Table 2 categorizes these desired tools and summarizes their perceived functions based on participant interviews.

**Table 2:** Potential roles of digital health technologies

Technology category	Specific tools/Examples mentioned	Primary uses/Perceived roles	Applicable domain	Related DSMES barriers
Health data Monitoring and tracking tools	Continuous glucose monitors (CGM) (e.g., Freestyle Libre), Standard Glucometers, Smartwatches (e.g., Apple Watch)	*Facilitating self-monitoring*: Providing real-time glucose data, reducing finger pricks.	SMB	-
		*Enhancing understanding*: Showing immediate impact of diet/activity.	SMB	-
		*Motivation & feedback*: Visualizing progress, prompting action.	SMB, DSMES	I. Perception that classes are non-interactive ii. Perceived lack of support
Mobile Apps, Web platforms, and social media	Diabetes education and support Apps, Websites (diabetes education websites, Google, YouTube, TikTok, Healthline, Facebook, Medical News Today), Digital Meal Planners/Recipe Finders, Digital Diaries/journaling, Artificial Intelligence (AI)-driven Feedback/Analysis, Integrated Data Visualization	*On-demand Information & Education*: Accessing articles, structured education materials, videos, recipes, verifying information.	DSMES, SMB	Competing demands
		*Tracking & Logging*: Recording food, activity, glucose, medication adherence.	DSMES, SMB	Perception that classes are non-interactive
		*Support & Community*: Connecting with peers, sharing experiences and personal journeys through social support chats.	SMB	-
		*Personalized guidance*: Assisting with meal planning, portion control, finding suitable recipes.	DSMES, SMB	Perceived lack of support
Telehealth & Communication	Video conferencing (Zoom), Telephone calls, Text messaging (SMS), Secure messaging (portals/apps), Email, AI Chatbots	*Remote Access*: Attending virtual classes/consultations.	DSMES	Competing demands, limited scheduling
		*Provider communication*: Asking questions, sharing data asynchronously.	DSMES, SMB	Perceived lack of support and follow-up
		*Advanced support*: Providing tailored suggestions/*feedback* based on logged data.	DSMES, SMB	Perceived lack of support, and follow-up
		*Reminders & nudges*: Receiving alerts for self-care tasks, appointments.	DSMES, SMB	Perceived lack of support, and follow-up

#### Theme 2: barriers to adoption across individual, technological, and system levels

Although participants identified the potential of digital health to facilitate engagement in DSMES and SMB, they also highlighted barriers operating at multiple levels. These are discussed under three subthemes.

##### Subtheme 2a: socioeconomic constraints

Financial barriers were pervasive and multifaceted. Participants noted that the high out-of-pocket costs of devices such as CGMs, health-tracking and planning apps, and online resources were significant obstacles to use. Although the cost of some devices, such as CGMs, may be covered by insurance for certain individuals, systemic barriers, including restrictive insurance eligibility criteria, were also identified. Other participants described challenges related to limited internet connectivity or the inability to afford smartphones compatible with newer technologies and applications. However, socioeconomic barriers did not necessarily reflect a lack of interest in digital health technologies. For example, one participant without a smartphone or stable internet access expressed a strong interest in using a CGM device. Another participant with prior CGM experience but limited financial resources similarly described a strong desire to continue using CGMs despite affordability challenges.

*“I really want help… find somebody that can supply those [CGMs] to me without paying all that money*,* I can’t really afford that”* (PWD3, never attended DSMES, a prior CGM user)

##### Subtheme 2b: limited digital literacy and usability challenges

Varying levels of digital literacy were also described as barriers to engagement. Some participants verbalized limited basic computer or smartphone skills, while others highlighted difficulties navigating health apps or patient portals that were overly complex or unintuitive. Participants suggested that these usability challenges could become overwhelming and discourage continued use.

“Yeah, basically just like making it easy to use…” (PWD1, never attended DSMES, owns a smartphone and attended the interview via Zoom)

“as simple as possible. So maybe icons or things you tap…” (Stakeholder 2)

##### Subtheme 2c: limited integration of digital tools into clinical care

Another important barrier identified by participants was the limited integration of digital tools into routine clinical care. Several participants reported being unaware of potentially useful apps or portals because their healthcare providers had not introduced or recommended them. Although some participants acknowledged the existence of communication portals within healthcare systems, many were unsure how to access or effectively use these platforms to communicate with their providers. Stakeholders also noted inconsistent provider use of existing digital communication tools, such as patient portals, and emphasized the need for greater provider education and familiarity with these platforms.

“…there also needs to be education on the provider side… if you ask me how to use the Athena [electronic health record] portal, I don’t know. I don’t use it” – Stakeholder 3

#### Theme 3: need for user-centered digital health solutions

Participants offered concrete recommendations for designing and implementing digital solutions tailored to the needs of low-income individuals newly diagnosed with T2D. Table 3 summarizes these key strategies.

**Table 3:** Key strategies recommended for user-centered digital solutions

Strategy category	Specific recommendations and sample quotes
Ensure affordability and accessibility	- Offer free or subsidized tools/devices/data plans. - Design for basic smartphones & varying connectivity levels. - Provide content in multiple languages, especially Spanish.
Prioritize simplicity and usability	- Create intuitive, easy-to-navigate interfaces - Avoid medical jargon; use plain language. Offer training and support for tool usage
Deliver personalized and relevant content	- Provide tailored meal plans, recipes, and nutrition guidance - Include personalized goal setting and feedback. - Offer curated, trustworthy educational resources (videos, articles). *“Maybe daily recommendations of what I can eat… I would want a timetable for a week"*
Incorporate engaging features	- Include robust health tracking functions (glucose, food, activity, medications). - Utilize reminders for self-care tasks & appointments. - Explore gamification or reward elements.
Facilitate communication and support	- Enable easy asynchronous communication with providers/educators (chat, secure messaging) - Integrate peer support features (forums, group chat, digital storytelling *“Being able to talk to other people with diabetes and share”* *“…it would be really cool if they could connect it to someone clinical who could answer if need be"*
Improve system integration and workflow	- Integrate digital tools with existing platforms (electronic health record systems (EHR), CGM apps) to reduce fragmentation - Ensure provider buy-in, training, and active promotion/use of tools - Streamline referral and scheduling processes involving digital components. *“…if there’s any way to layer it over the EHR or a CGM or existing platforms*,* that might be the best”*.

## Discussion

This qualitative study explored barriers and facilitators to DSMES engagement among low-income adults newly diagnosed with T2D, as well as the potential role of digital health technologies in supporting DSMES participation and SMBs. By centering adults’ experiences early in the diagnosis period, this study addresses a critical yet underexamined stage when engagement with DSMES is essential yet often delayed or missed. A major finding was the importance of the psychological adjustment period following diagnosis, during which participants described denial, apathy, anxiety, and emotional overwhelm that reduced readiness to engage in DSMES or initiate SMBs. While prior research [22, 23] has largely emphasized structural and programmatic barriers, including transportation difficulties, competing responsibilities, inflexible scheduling, and limited referrals, our findings suggest that these challenges often interact with psychosocial distress to further impede engagement. Together, these findings highlight the need for early diabetes care approaches that integrate accessible DSMES referral pathways, supportive provider communication, and brief psychosocial assessment and support into routine clinical encounters immediately following diagnosis.

Our findings indicate that low-income adults newly diagnosed with T2D value DSMES approaches that are flexible, continuous, and responsive to the emotional and practical disruptions that often follow diagnosis. Rather than viewing DSMES as a one-time educational encounter, participants appeared to value sustained access to guidance and communication that could be integrated into daily life. Digital approaches such as telehealth, asynchronous educational platforms, mobile applications, and messaging tools may therefore help address not only logistical barriers to DSMES participation but also the uncertainty and emotional distress that may interfere with early self-management efforts. Prior research has shown that online DSMES and digital education can improve self-management behaviors and glycemic outcomes across diverse T2D populations [24, 25]. Extending this literature, digitally delivered education and communication may reinforce engagement in SMBs by improving access to timely information, behavioral guidance, and interpersonal connection during the early post-diagnosis period [16, 17]. These findings also align with the Information-Motivation-Behavioral Skills model, which posits that individuals require adequate information, motivation, and behavioral skills to initiate and sustain health behaviors. Digital DSMES tools may therefore support engagement by strengthening behavioral skills and motivation while improving access to self-management education.

At the same time, participants and stakeholders consistently emphasized that in-person DSMES remains an essential component of early diabetes care, particularly for newly diagnosed individuals requiring foundational knowledge and support. This suggests that digital solutions are likely to be more effective when implemented as a supportive extension of, not a replacement for, in-person DSMES. Rather than suggesting a single comprehensive digital platform, our findings indicate that different technologies may serve complementary roles across DSMES engagement and SMB support. Telehealth may improve access to education, communication platforms may facilitate ongoing support, and monitoring technologies such as CGMs may reinforce behavioral awareness and self-management. Stakeholders additionally noted that integrating digital tools into DSMES sessions could strengthen patient-provider interactions through features such as data visualization and ongoing feedback. Collectively, these findings support hybrid care models that combine digital technologies with in-person DSMES delivery to improve flexibility, continuity of support, and long-term engagement. Future research should identify which combinations of digital tools are most feasible, acceptable, and sustainable for supporting DSMES engagement among low-income adults newly diagnosed with T2D. Importantly, however, participants and stakeholders also identified system-level implementation challenges, including competing clinical demands, limited workflow integration, and insufficient provider training, which may hinder sustained use of digital DSMES tools. Successful implementation will therefore require not only patient-facing interventions but also provider training and integration of digital platforms into routine clinical workflows.

Despite the promise of digital DSMES approaches, our findings highlight persistent equity-related and policy-level challenges that may limit their reach and effectiveness among underserved populations. Affordability emerged as a major concern, reflecting the broader digital divide that continues to shape access to digital health resources [26]. Consistent with prior literature [26, 27], our findings suggest that financial barriers may disproportionately restrict access to technologies intended to improve diabetes self-management and support. Importantly, affordability barriers should not be interpreted as a lack of interest in digital health technologies among low-income populations. In fact, some participants facing the greatest financial constraints expressed strong interest in technologies such as CGMs, despite limited ability to afford them. Without deliberate efforts to address affordability and access, digital DSMES interventions may unintentionally widen existing disparities in diabetes care.

Policy-level barriers remain important, particularly inconsistent reimbursement parity between telehealth and in-person DSMES services across states. Variability in telehealth policies, including restrictions related to eligible providers, approved communication formats, and coverage requirements, may limit the scalability and sustainability of digital DSMES delivery models [28]. Arizona, where this study was conducted, is among the states that have enacted reimbursement parity for telehealth and in-person services, potentially creating a more supportive environment for digital DSMES implementation [28]. Continued national advocacy for reimbursement parity, broader telehealth coverage, and low-cost device and internet access programs may therefore be necessary to support equitable access and broader adoption of digital DSMES approaches among underserved populations.

Beyond financial and policy-level barriers, participants’ concerns regarding complex or unintuitive platforms suggest that access to technology alone is insufficient to support meaningful and sustained engagement. These findings underscore the importance of integrating co-creation and community-based participatory research approaches early in the development of digital DSMES interventions to ensure that tools are culturally relevant, responsive to the lived experiences of underserved populations, adaptable to varying literacy levels, and easy to navigate. Prior evidence similarly highlights the value of participatory and culturally sensitive approaches in designing equity-focused diabetes self-management interventions for underserved populations [29]. Broader digital health literature suggests that co-design approaches may improve the relevance, usability, and sustainability of digital interventions [30]. Given prior evidence that engagement with digital health interventions often declines over time [31], future research should additionally examine how some of these participatory approaches can support sustained participation and long-term use of digital DSMES tools in real-world settings.

Finally, although participants largely described digital monitoring technologies such as CGMs and tracking applications as motivating and empowering for behavioral change, our findings also highlight the potential unintended consequences and differing perspectives regarding their use. One participant characterized CGM use as a “self-invented intervention” that increased awareness of how lifestyle behaviors affected blood glucose and self-management, whereas one stakeholder expressed concern about information overload or anxiety from continuous health data. This discrepancy may reflect differing perceptions between healthcare providers and patients regarding the value and emotional impact of self-monitoring technologies. Rather than suggesting that these tools are inherently beneficial or harmful, our findings underscore the importance of patient-centered approaches that account for individual preferences, emotional responses, and readiness to engage with data. Patient-centered approaches are widely recommended to improve healthcare experiences, engagement, and health outcomes [7] and may be particularly important when recommending digital technologies for newly diagnosed individuals with varying psychosocial needs and readiness for self-management. Some participants also raised concerns regarding privacy and data security, highlighting the need for transparent communication and clear safeguards when integrating digital technologies into DSMES platforms.

### Healthcare and policy recommendations

Healthcare providers should prioritize the timely referral of low-income adults newly diagnosed with T2D to DSMES to address early psychosocial, educational, and self-management support needs. Providers should also proactively follow up with referred individuals to understand their experiences, identify barriers to engagement, and address emotional concerns that may affect DSMES participation and retention. Healthcare providers and diabetes educators should consider hybrid DSMES models that thoughtfully integrate evidence-informed digital health tools, such as asynchronous learning platforms, health-monitoring technologies, communication platforms, and social support features, to improve accessibility, engagement, and ongoing support. Healthcare systems should additionally invest in provider training and workflow integration strategies to support effective implementation and sustained use of digital DSMES tools.

From a policy perspective, equitable implementation of digital DSMES will require coordinated efforts across reimbursement, coverage, and digital infrastructure. Expanding reimbursement parity for telehealth and digitally delivered DSMES services across Medicare, Medicaid, and commercial insurance plans may help reduce financial barriers that limit program availability and uptake among underserved populations. Continued investment in broadband infrastructure and low-cost internet access programs is also essential to addressing connectivity barriers that constrain participation in telehealth-based DSMES. Collectively, these recommendations underscore the need for coordinated action across clinical practice, technology design, and policy to ensure that digital health solutions support equitable DSMES engagement and sustained diabetes self-management outcomes.

### Limitations

This study has several limitations. Its qualitative nature provides depth of understanding but limits generalizability, as the findings reflect the perspectives of a specific low-income population and stakeholder group within a single geographic region. Although the study focused on adults newly diagnosed with T2D, it also included individuals who had lived with diabetes for longer periods to capture broader perspectives on DSMES engagement and digital health use across the disease trajectory. We acknowledge that the inclusion of participants diagnosed many years earlier may have introduced recall bias regarding experiences at the time of diagnosis. In addition, all interviews were conducted in English. Although many Hispanic/Latino participants were bilingual in English and Spanish, the study may have excluded individuals who primarily speak Spanish or have lower literacy levels, thereby limiting representation of populations who may face additional barriers to DSMES participation and digital health engagement. Finally, while this study focused on T2D, the findings may not fully apply to individuals with type 1 diabetes, who may have distinct self-management needs, technology use patterns, and access challenges.

## Conclusions

DSMES remains a critical evidence-based intervention for individuals newly diagnosed with T2D, yet engagement is often delayed by emotional distress, uncertainty, competing life demands, and limited access to timely support during the early post-diagnosis period. Our findings suggest that digital health technologies, including telehealth, asynchronous learning, remote monitoring, and communication platforms, may help improve access to diabetes education and ongoing support for low-income adults newly diagnosed with T2D. Hybrid approaches that integrate digital tools with in-person DSMES may be particularly valuable for providing flexible, continuous, and patient-centered support while strengthening engagement in self-management behaviors. To ensure equitable benefit, digital DSMES approaches should be affordable, easy to navigate, responsive to users’ needs, integrated into routine care delivery, and supported by enabling policies.

## Supplementary Information

Supplementary Material 1.

Supplementary Material 2.

Supplementary Material 3.

## Data Availability

The corresponding author will provide data related to this manuscript upon considerable request.

## Abbreviations

T2D: Type 2 diabetes
SMB: Self–management behaviors
DSMES: Diabetes self–management education and support
USA: United States of America
CGM: Continuous glucose monitor
SMS: Short Message Service
COREQ: Consolidated Criteria for Reporting Qualitative Research
PI: Principal Investigator
PWD: Person with diabetes
AI: Artificial Intelligence
EHR: Electronic Health Record
