# Supplementary material for: Perceived role of digital health technologies for facilitating diabetes self-management and education among low-income adults with type 2 diabetes: a qualitative needs assessment to inform support for newly diagnosed adults
Source: BMC Public Health. 2026 Aug 11;26:3229. doi: 10.1186/s12889-026-28880-2 (PMC13741496; doi:10.1186/s12889-026-28880-2)
Supplement: Supplementary file 2 [file 12889_2026_28880_MOESM1_ESM.docx]

Interview guide

1. What do you think are the greatest needs for low-income people when they are newly diagnosed with T2D and as they continue to manage the disease?
2. How do you think the healthcare system can better support people when they are newly diagnosed with T2D or struggling to manage their diabetes?
3. What do you think the community can do to support people better when they are newly diagnosed with T2D or struggling to manage their diabetes?
4. What do you think the government can do to better support people when they are newly diagnosed?
5. Do you refer people for DSMES (Explain DSMES)?
6. What are some of the challenges you have experienced, or that your patients have encountered or complained about?
7. What are some things we can do to better engage them in DSME?
8. Do you think sending reminders before patients’ appointments, reminding them of what they learned in the class, and reminding them of the goals they set would be helpful?

- If yes, what do you see as potential challenges?
- If not, why not? What would work better?

9. What kinds of technology (give examples such as mobile apps, websites, continuous glucose monitoring, text messaging) do you think people with type 2 diabetes use the most

- Which ones do you think will be the most useful form of technology for them?

10. What are the key features that a technology/digital platform designed for low-income people who are newly diagnosed or with poorly controlled diabetes should have?

Closing

Is there anything you would like to share?
