# Supplementary material for: Perceived role of digital health technologies for facilitating diabetes self-management and education among low-income adults with type 2 diabetes: a qualitative needs assessment to inform support for newly diagnosed adults
Source: BMC Public Health. 2026 Aug 11;26:3229. doi: 10.1186/s12889-026-28880-2 (PMC13741496; doi:10.1186/s12889-026-28880-2)
Supplement: Supplementary file 3 [file 12889_2026_28880_MOESM2_ESM.docx]

**Interview guide for people with type 2 diabetes**

**Overview of the purpose of the interview**

The goal of this interview is to help us learn how to better support people living with type 2 diabetes, beginning from when they are diagnosed. We will ask you about what it is like to manage your diabetes – what makes it easier, and what makes it harder.

Additionally, we are interested in the kinds of information, resources, or support you feel are most helpful for effective diabetes management, how best to provide that information and support, and what you think a diabetes education class should include.

We’ll conclude by discussing your thoughts on apps on your phone and other technologies that could help people manage their diabetes.

| **Purpose/Framework target** | **Interview questions** |
| --- | --- |
| Icebreaker/Diagnosis | 1. Can you tell me about when you first learned you had diabetes?  - How did you find out? - How was the diagnosis explained to you? - What was it like to receive this news? - What was your initial reaction? |
| Icebreaker/coping | 2. After reflecting on your diagnosis, can you tell me what it feels like to live with diabetes now?  - How does it affect you? - How has it changed how you see yourself? - How does it affect your mood or feelings? - How does it change your day-to-day activities and work? - How does it impact your social life and interactions with others? - How does it impact other people around you (family, friends)? |
| Diabetes self-management/existing information/knowledge | 3. How do you manage or take care of your diabetes?  - What are some lifestyle changes you have made? - What medical help are you getting? |
| Diabetes self-management/information source(s) | 4. Thinking about the things you do to take care of your diabetes, how did you learn about them?  - Did you learn about them from other people (who are they)? - Did you find information online? If so, where? - Did you go to any classes? |
| Information and skill needs | 5. What else would you like to learn to help you take care of your diabetes better?  - Are there specific topics you’d like to learn more about? - Are there any practical skills you’d like to learn? |
| Newly diagnosed | 6. Imagine a friend just found out they have diabetes, but they don’t have insurance or much money. What kinds of programs, services, or support would help them get started with their care? |
| **Transition to DSMES** | **Script:** Now let’s shift gears a bit. I’d like to talk about a program called Diabetes Self-Management Education and Support (DSMES for short). This program teaches people about type 2 diabetes and provides them the information and skills they need to take care of themselves. Sometimes, people also go to other classes, like a one-time nutrition class, which are different from DSMES. When you answer, please keep in mind which type of program you’re talking about. |
|  | 7. What do you know about DSMES?  - How did you first hear about it? - What do you think this program is or what it offers? |
| DSMES awareness | 8. Have you ever taken part in DSMES or any other diabetes classes?   **If YES**:   - What was the format and structure of the class? (e.g., online/in-person/group/individual, number of classes)? - How did you get into the program?   **If NOT a standard DSMES**   - Can you tell me about the program? - What was the format - What was the purpose - What was the frequency? |
|  | **Skip 9-13, go to Q14 if participant did not attend DSMES class** |
| Enrollment | 9. Can you tell me what made you decide to go to the classes?  - Was it suggested by a friend? - Was it suggested by your provider? - Was it because of your diabetes situation at the time? |
|  | 10. How long did it take before you started your first class after being referred?  - If there was a delay, what caused it? - What was the process like from referral to showing up? - What were the things that enabled you to remember your appointment? - What challenges came up along the way? |
| **Attendance** | 11. How would you describe your attendance?  - How many classes did you attend? - What made it easy or hard to attend? - What would make it easier to attend as required? |
| Experience | 12. What was your experience in the classes like?  - What did you learn? - What stood out to you? - What did you enjoy most? - Did the program meet your expectations? |
| Improvement opportunities | 13. What could the program do to make the experience better for you? |
| **Non-attendance** | 14. Can you tell me why you didn’t go to the classes?” |
|  | 15. Attending all the classes can be hard. What are some things that make it difficult for people? |
|  | 16. What do you think would help people sign up, attend the classes, and enjoy them?  - What might especially interest someone who just found out they have diabetes and doesn’t have many resources? |
| **Transition to digitally enhanced self-management and DSMES** | **Script:** *We are now in the final section of the interview, where we will discuss how digital technologies can help you manage your diabetes. When we talk about digital technologies, think about things like: mobile/smart phones, text messaging, emails, AI chatbots, websites, apps, patient communication portals, game trackers, etc. These are programs or apps that work on your phone, computer, or social media.* |
|  | 17. Tell me about the kinds of technology you use to help manage your diabetes  - What is the tool, and what do you use it for? - How did you hear about it, and what made you decide to get it? - How did you get access to it? - What do you find most helpful about it? - Is there anything about it that isn’t helpful?” |
|  | 18. What other digital tools do you think would be useful to help you manage your diabetes? And why? |
|  | 19. If we created a new digital program to help people manage diabetes, what would you want it to be like?  - What kind of platform would work best for you? A website, an app, text messages, a chatbot, a patient portal, or something else?   - Why would that be the best option? - What would make you want to use a digital program for diabetes? - What kinds of functionality should it have, e.g., should it include videos, audio, or other features? - What kind of content would you like? For example, should it cover stress? |
|  | 20. What might turn you off from using a digital tool for diabetes?  - The wording it uses? - Who the program is linked to? - Certain topics covered? - Privacy or trust issues? |
| **Digitally enhanced DSMES** | **Script:** *We are in the last section. We will be talking about the role of digital health technology in improving DSMES experience.* |
|  | 21. How could digital tools make diabetes classes (DSMES) a better experience for patients?  - Think about ways technology could add to the classes – for example, reminders, online resources, videos, apps, or ways to connect with others. |
|  | 22. How could digital tools help people attend, stay engaged in, and complete DSMES classes?  - Think about things like reminders, interactive content, peer support, or rewards that might keep people involved. |
| **Digital health barriers** | 23. What concerns or challenges should we think about when using digital tools for diabetes self-care or for improving classes? |
|  | 24. For individuals with limited resources and financial difficulties, what digital tools do you think would be most useful? What digital tools would be less useful? |
| **Closing** | We’ve come to the end of the interview. Thank you so much for sharing your story with me. Is there anything else you’d like to share? We are also planning a focus group discussion for this study later on. Is it okay if we contact you to see if you would like to be part of it? |
